# Examining the effects of uric acid-lowering on markers vascular of calcification and CKD-MBD; A post-hoc analysis of a randomized clinical trial

**DOI:** 10.1371/journal.pone.0205831

**Published:** 2018-10-24

**Authors:** Emily S. Andrews, Loni Perrenoud, Kristen L. Nowak, Zhiying You, Andreas Pasch, Michel Chonchol, Jessica Kendrick, Diana Jalal

**Affiliations:** 1 Division of Renal Diseases and Hypertension, Department of Medicine, University of Colorado Anschutz Medical Campus, Aurora, CO, United States of America; 2 Department of Biomedical Research, University of Bern, Bern, Switzerland; 3 Nephrology Division, The University of Iowa, Iowa City, IA, United States of America; Universidade Nove de Julho, BRAZIL

## Abstract

**Background:**

Chronic kidney disease (CKD)-mineral and bone disorder (MBD) is a systemic disorder that leads to vascular calcification and accelerated atherosclerosis. Uric acid has been shown to associate with vascular calcification and with carotid intima-media thickness (CIMT) and to suppress the 1 α-hydroxylase enzyme leading to lower 1,25-dihydroxyvitamin D (1,25(OH)2D) and higher intact parathyroid hormone (iPTH) levels. We hypothesized that lowering serum uric acid would reduce CIMT, calcification propensity, and circulating markers of CKD-MBD in CKD.

**Methods:**

This is a post-hoc analysis of a randomized, double-blind study of 80 patients with stage 3 CKD and hyperuricemia who received allopurinol or placebo for 12 weeks. CIMT and T_50_ were measured as markers of vascular disease and serum calcification propensity, respectively. The following markers of CKD-MBD were measured: serum calcium, phosphorus, vitamin D metabolites, iPTH, and fibroblast growth factor-23 (FGF-23). Expression of extra-renal 1α-hydroxylase was evaluated in endothelial cells of study participants.

**Findings:**

Allopurinol successfully lowered serum uric acid levels compared to placebo with an estimate of -3.3 mg/dL (95% C.I. -4.1,-2.5; p < 0.0001). After 12 weeks, however, we found no significant change in CIMT or serum T_50_. There was not a significant change in vitamin D metabolites, iPTH, FGF-23, or the expression of endothelial 1α-hydroxylase.

**Conclusion:**

These data suggest that factors other than uric acid may play a more important role in the regulation of CKD- MBD including vascular calcification and vitamin D metabolism in patients with CKD.

## Introduction

Chronic kidney disease (CKD) is characterized by accelerated vascular aging and atherosclerosis of which vascular calcification is a dominant feature [1–3]. It is induced by dysregulation of the mineral and bone axis (CKD-associated mineral and bone disorder) as well as by local inflammation, elastin degradation, and vascular smooth muscle osteogenic differentiation [4–6]. A systemic complication of CKD, vascular calcification may involve the intima, the media, or both [7] and is predictive of cardiovascular events in this patient population [8]. In addition, vascular calcification has been reported to correlate with surrogate outcomes of cardiovascular disease (CVD) such as carotid intima-media thickness (CIMT) [9], an early marker of atherosclerosis and an established risk factor for CVD [10, 11].

CKD-associated mineral and bone disorder (CKD-MBD) is a complex disorder encompassing a wide range of abnormalities in mineral and bone metabolism including calcium, phosphorus, vitamin D, parathyroid hormone, and fibroblast growth factor (FGF)-23 [12]. Collectively, these abnormalities are believed to contribute to vascular calcification and accelerated atherosclerosis in CKD. Uric acid is the main end-product of purine metabolism in humans [13] and it is filtered and, subsequently, reabsorbed and secreted by the kidney [14]. Generally, the pathological changes caused by uric acid, such as gout and kidney stones, are the consequences of its insolubility at higher concentrations whereby a high uric acid level induces urate crystallization in multiple organs [15]. It has recently been postulated that hyperuricemia is in the causal pathway of hypertension, CKD progression, and cardiovascular disease (CVD) by non-crystal mechanisms [14]. The prevalence of hyperuricemia is, in fact, increased in patients with CKD and has been linked in epidemiological studies to both CKD progression and CVD in patients with CKD [14].

Previously published data by our group have linked hyperuricemia to CKD-MBD, as we have shown uric acid suppresses 1α-hydroxylase expression and activity *in vitro* in renal proximal tubular cells and *in vivo* in a rodent model of acute hyperuricemia [16]. We have also shown increased uric acid levels associate with increased intact parathyroid hormone (iPTH) levels independently of the degree of CKD [16]. We have noted hyperuricemia associates with an increased risk of fractures in elderly community-dwelling adults [17] and with coronary calcification in patients with type 1 diabetes mellitus [18]. Other groups have published similar findings associating asymptomatic hyperuricemia with coronary calcification [19, 20] and with CIMT [21, 22]. Based on these data, we hypothesized that lowering serum uric acid levels would improve CKD-MBD parameters and slow vascular calcification in patients with CKD. We evaluated this in a post-hoc analysis of a recently published randomized, double-blind, placebo-controlled clinical trial of the effects of uric acid-lowering on vascular endothelial dysfunction in patients with stage 3 CKD and hyperuricemia [23]. We utilized CIMT and T_50_ (a serum calcification propensity score shown to predict mortality in CKD) as markers of vascular calcification [9, 24].

## Methods

### Study population

The details of the parent study have been published previously [23]. Briefly, to qualify for participation, male patients were required to have a serum uric acid level of ≥7.0 mg/dL and female patients a serum uric acid level of ≥6.0 mg/dL with stage 3 CKD (eGFR between 30–60 mL/min/1.73^2^) calculated based on the Modification of Diet in Renal Disease (MDRD) formula [25]. Additionally, patients had to be 18–74 years of age, have a serum albumin >3.0, and be able to give informed consent. Exclusion criteria included: any condition that would contraindicate use of allopurinol such as severe liver disease, a history of severe congestive heart failure, any infection within the past 2 weeks, any hospitalization within the past 3 months, use of coumadin, and a body mass index >40 kg/m^2^. Additionally, patients were excluded from participation if they were currently taking allopurinol or another uric acid-lowering agent or if they had a history of intolerance to allopurinol. The study was approved by the Colorado Multiple Institutional Review Board and all participants were required to provide written consent prior to participation in the study. For purposes of this analysis, we included only participants, who had provided written consent for the future use of their samples and had adequate sample volumes (n = 63). The study was registered at ClinicalTrials.gov, NCT01228903.

### Study intervention

Patients were randomly assigned to receive allopurinol or placebo over a 12-week period and were advised to take 100 mg/day during the first week, 200 mg/day during the second week, and 300 mg/day for weeks 3–12.

### Assessments

*- Carotid- intima-media thickness (CIMT)*: was measured in a double-blind manner at the Clinical and Translational Research Center (CTRC) at the University of Colorado Anschutz Medical Center. Briefly, the left common carotid artery was imaged via GE Vivid 7 ultrasound equipped with a linear array transducer and carotid artery diameter was analyzed using image analysis software (Carotid Analyzer version 5.10.10, Medical Imaging Applications) as previously described [26]. A longitudinal segment of the cephalic portion of the carotid artery was acquired ~2 cm distal to the carotid bulb for at least 10 cardiac cycles. Carotid IMT was defined as the distance from the leading edge of the lumen-intima interface to the leading edge of the media-adventitia interface on the far wall, measured during end diastole. All image analysis was performed by a single investigator blinded to the study randomization.

- T_50_: We utilized a novel *in vitro* blood test to measure the overall calcification propensity by monitoring calciprotein particles (CPPs) maturation time (T_50_). This measurement, as previously described [27], is a nanoparticle-based assay that detects the spontaneous transformation of spherical colloidal primary CPPs to elongate crystalline secondary CPPs in the presence of artificially elevated calcium and phosphate concentrations. Lower (compared to higher) T_50_ values have been shown to predict all-cause mortality in patients with CKD [24]. The samples were measured for T_50_ in the clinical research unit at Bern University.

- CKD-MBD markers: Analysis of 1,25(OH)_2_D was performed on frozen serum samples using immunoaffinity extraction and high-performance liquid chromatography (HPLC) mass spectrophotometry (XEVO TQ, Waters) at the University of Washington’s Laboratory for Nutrition and Obesity Research Center. The detailed methodology and assay optimization has been previously published [28]. The method has lower limits of quantification (20% coefficient of variation) of 1.25 pg/ml for 1,25(OH)_2_D_3_ and 0.64 pg/ml for 1,25(OH)_2_D_2_. Published interassay and intraassay imprecision for this method are <14% for both 1,25(OH)_2_D_3_ and 1,25(OH)_2_D_2_. Total 1,25(OH)_2_D is reported as the sum of 1,25(OH)_2_D_3_ and 1,25(OH)_2_D_2_. The subset of measured samples consisted of samples stored at -80°C. Specifically, 29 samples were available from the allopurinol group and 34 from the placebo group. We were unable to evaluate all the participants from the larger parent study involving 80 patients due to lack of adequate sample volume. To reduce the number of thaw-refreeze cycles, 25(OH)D and 24,25(OH)_2_D were measured simultaneously utilizing the same analysis and extraction procedure [29, 30]. The interassay coefficients of variation were <3.4% and 14.7%, respectively. [31] FGF-23 and iPTH analyses were also conducted at the University of Washington laboratories using the same frozen serum samples. Full-length (intact) FGF-23 levels were measured via sandwich ELISA assay (Kainos, Japan) [31]. The interassay coefficient of variation (CV) = 12.4%. iPTH levels were measured by using a 2-site immunoassay via a Beckman Unicel DxI clinical analyzer [31]. The interassay CV = 6.1%. Calcium and phosphorus were analyzed using ambient plasma samples on the day of collection by the University of Colorado Hospital clinical lab.

- Endothelial cell expression of 1α-hydroxylase: This was evaluated as a measure of extra-renal 1α-hydroxylase using endothelial cells collected from the antecubital vein of study participants as previously published [32, 33]. Briefly, immediately after cell collection, cells were fixed with 3.7% formaldehyde, washed with 1x PBS, and plated onto slides. Cells were then blocked with 5% Normal Donkey Serum (Jackson Immunoresearch) and incubated with primary antibody (The Binding Site) for 1 hour. After, cells were incubated with CY3-congugated secondary antibodies (Life Technologies) for 45 minutes. Slides were scanned systematically to identify cells with intact nuclei; intact nuclei were identified by using 4',6'-diamidino-2-phenylindole hydrochloride (Vector Laboratories). Endothelial cells were then identified by positive VE-Cadherin (Adcam) staining. Once intact endothelial cells were identified, the images were captured and analyzed using NIS Elements AR software (Laboratory Imaging). An average pixel intensity of 30 intact endothelial cells was used to quantify the intensity of the Cy3 staining. These values were then reported as a ratio of endothelial cell protein expression to human umbilical vein endothelial expression. This ratio was used to account for any variation in the staining procedure that may occur. Of note, 1α-hydroxylase was evaluated in 28 subjects; placebo (n = 8) and allopurinol (n = 20), all by the same analyst. The small number of samples is due to the inadequate number of cells on some of our collections or to some patients not undergoing the sample collection.

### Statistical analysis

Baseline characteristics by group are presented as mean ± SD for continuous variables with categorical variables shown as %. Variables that are not normally distributed were transformed. We conducted the analysis utilizing generalized linear mixed (GLM) modelling. We evaluated the distribution of the outcomes including CIMT, T_50_, 25(OH)D, 24,25(OH)_2_D, and 1,25(OH)_2_D, FGF-23, and iPTH. The majority had normal distribution so we ran mixed models using a normal distribution. The only exception was for FGF-23, for which Gamma distribution with a log link function was used. There was one value that was an outlier for each: CIMT and FGF-23 (significantly larger than the others) and they were both recoded to missing for the modeling. A random intercept was included for each outcome. A random residual was included for each outcome except for 24,25(OH)2D. In addition to the unadjusted analysis, we adjusted for age, sex, race/ethnicity. We applied the same approach to analysis of 1α-hydroxylase protein expression in the endothelial cells. SAS software (version 9.4, Cary, N.C.) was used to conduct all analyses.

## Results

### Clinical characteristics at baseline

Patient baseline characteristics are presented in Table 1. The majority of patients in both groups were male but there were more men in the treatment arm (79% in the placebo group and 83% in the allopurinol group). Additionally, the patients were predominantly Caucasian with a higher % in the placebo group (85% compared to 62% in the allopurinol group). Over half of the patients in each group had a history of DM with 62% of those assigned to the placebo and 66% of those assigned to allopurinol being diabetic. Chronic Kidney Disease Epidemiology Collaboration (CKD-EPI) estimated GFR [34] was 40.8 ± 8.4 mL/min/1.73^2^ in the placebo group and 40.3 ± 9.3 mL/min/1.73^2^ in the allopurinol group. Serum uric acid levels nor MBD markers were significantly different between the groups at baseline except for FGF-23, which was quantitatively lower in the group randomized to allopurinol compared to placebo (p value = 0.002).

**Table 1:** Baseline characteristics of the subset of subjects with CKD-MBD measurements according to study group.

	Placebo (n = 34)	Allopurinol (n = 29)
**Age (years)**	58.0 ± 9.6	59.2 ± 12.9
**Gender (Male)**	27 (79%)	24 (83%)
**Race**		
Caucasian	29 (85%)	18 (62%)
African American	3 (9%)	7 (24%)
Other	2 (6%)	4 (14%)
**Hispanic ethnicity**	9 (27%)	5 (17%)
**History of DM**	21 (62%)	19 (66%)
**CKD-EPI eGFR (mL/min/1.73m**^**2**^**)**	40.8 ± 8.4	40.3 ± 9.3
**Serum urate (mg/dL)**	8.81 ± 1.4	8.39 ± 1.4
**CIMT (mm)**	0.77 ± 0.17	0.73 ± 0.14
**Serum T**_**50**_ **(min)**	274 ± 56	244 ± 53
**Calcium (mg/dL)**	9.4 ± 0.4	9.1 ± 0.4
**Phosphorus (mg/dL)**	3.6 ± 0.6	3.6 ± 0.5
**25 vitamin D (ng/mL)**	29.4 ± 10.1	26.8 ± 8.1
**Total 1,25 vitamin D (pg/mL)**	28.0 ± 12.0	27.2 ± 11.0
**24,25 vitamin D (pg/mL)**	3.4 ± 1.9	3.2 ± 1.8
**iPTH (pg/mL)**	73.8 ± 40.8	68.4 ± 32.4
**FGF-23 (pg/mL)-Median (IQR)**	110.4(92.7,150.4)	74.1(57.3,104.9)*

Values are expressed as means ± standard deviation or % = percent of patients unless otherwise noted iPTH = intact parathyroid hormone; FGF-23 = fibroblast growth factor-23

*: p value <0.05

### The effects of uric acid-lowering on CIMT and serum calcification propensity

Baseline CIMT and T_50_ were similar between both groups at baseline. Across the 12 weeks of the study, allopurinol successfully lowered serum uric acid levels compared to placebo with an estimate of -3.3 mg/dL (95% C.I. -4.1,-2.5; p < 0.0001). There was no significant change in CIMT over a period of 12 weeks in either the treatment or the placebo group. The estimate was -0.02 (95% C.I. -0.06, 0.02, p = 0.43). As shown in Table 2, we did not observe a significant change in T_50_ between the group treated with allopurinol vs the group treated with placebo. Of note, adjustment for sex and race/ethnicity did not alter our findings (data not shown). Additionally, there was not a significant interaction with eGFR, neither as a continuous variable nor using a cut-off of 45 mL/min/1.73m^2^, and CIMT or T_50_. We, furthermore, found no significant change in the participants’ fasting glucose or lipid profile (data not shown).

**Table 2:** Effect of treatment group compared to placebo over the 12-weeks study period.

Variable	Estimate for Treatment group*Time	95% C.I.	p value
**Serum urate (mg/dL)**	-3.3	-4.1, -2.5	<0.0001
**CIMT (mm)**	-0.02	-0.06, 0.02	0.43
**Serum T**_**50**_ **(min)**	-11.4	-38.3, 15.5	0.41
**Calcium (mg/dL)**	0.04	-0.15, 0.23	0.67
**Phosphorus (mg/dL)**	0.15	-0.14, 0.44	0.32
**25 vitamin D (ng/mL)**	-2.12	-4.94, 0.70	0.15
**Total 1,25 vitamin D (pg/mL)**	-2.47	-7.08, 2.14	0.30
**24,25 vitamin D (pg/mL)**	-0.29	-0.66, 0.08	0.14
**iPTH (pg/mL)**	0.28	-14.9, 15.5	0.97
**FGF-23 (pg/mL)**	0.13	-0.05, 0.31	0.17

iPTH = intact parathyroid hormone; FGF-23 = fibroblast growth factor-23; CIMT = carotid intima-media thickness

*: interaction

### The effects of uric acid-lowering on vitamin D metabolites and other CKD-MBD parameters

Table 2 illustrates that there was no effect for treatment group over the 12-weeks study period on 25(OH)D, 24,25(OH)_2_D, and 1,25(OH)_2_D. Additionally, no significant effect of treatment versus placebo was found on mineral and bone markers including serum calcium, phosphorus, or iPTH. FGF-23 levels did increase slightly for the group that received allopurinol but this did not reach statistical significance (estimate = 0.13 with 95% C.I. -0.05, 0.31; p = 0.17). Of note, further adjustment for sex and race/ethnicity did not change our results (data not shown). Furthermore, we found no significant interaction in any of our observations for CKD-MBD parameters and eGFR.

### Uric acid-lowering and the expression of 1α-hydroxylase in the endothelium of study participants

**Fig 1:**
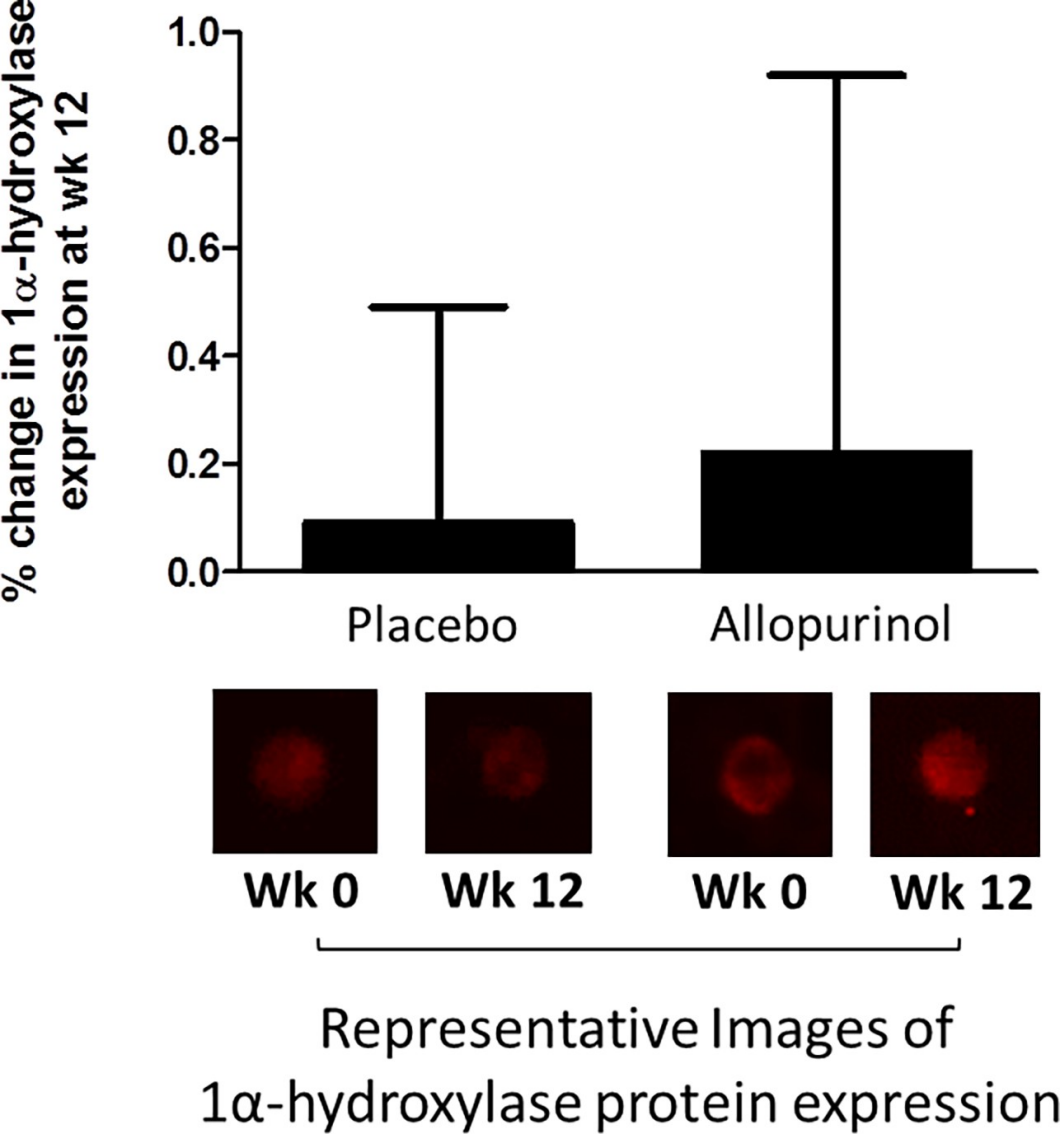
Treatment with allopurinol for 12 weeks was not associated with increased expression of 1α- hydroxxylase protein compared to placebo (p value for generalized linear mixed model = 0.59). The figures represent change in the expression of the protein from baseline by study group. Values for each sample were reported as arbitrary units and represent ratios of endothelial cell protein expression to human umbilical vein endothelial cell (HUVEC) expression in order to account for any variation in the staining procedure.

The clinical characteristics for the participants with endothelial 1α-hydroxylase expression are shown in Table 3. As shown in Fig 1, baseline expression of 1α-hydroxylase protein was similar between the patients who were randomized to allopurinol vs those who received placebo at baseline. In evaluating whether treatment arm influenced the expression of the 1α-hydroxylase protein in the endothelial cells of participants over the 12-weeks study period, we found no significance (p value = 0.59).

**Table 3:** Baseline characteristics of the subset of subjects with 1α-hydroxylase protein expression according to study group.

	Placebo (n = 8)	Allopurinol (n = 20)
**Age (years)**	57.5 ± 9.4	54.8 ± 15.7
**Gender (Male)**	4 (50%)	8 (66.7%)
**CKD-epi eGFR (mL/min/1.73m**^**2**^**)**	41.4 ± 12.4	39.0 ± 11.2
**Calcium (mg/dL)**	9.3 ± 0.3	9.0 ± 0.4
**Phosphorus (mg/dL)**	3.5 ± 0.7	3.5 ± 0.8
**25 vitamin D (ng/mL)**	3.3 ± 0.3	3.2 ± 0.2
**Total 1,25 vitamin D (pg/mL)**	3.1 ± 0.5	3.1 ± 0.5
**24,25 vitamin D (pg/mL)**	0.9 ± 0.7	0.4 ± 1.2
**iPTH (pg/mL)**	83.6 ± 38.7	81.0 ± 24.2
**FGF-23 (pg/mL)-Median (IQR)**	110.4 (28.9, 470.4)	74.1 (28.1, 151.2)
**1α-hydroxylase**	0.7 ± 0.3	0.8 ± 0.2

Values are expressed as means ± standard deviation, median (interquartile range), or % = percent of patients; iPTH = intact parathyroid hormone; FGF-23 = fibroblast growth factor-23

## Discussion

In this study, we evaluated whether lowering serum uric acid levels in patients with stage 3 CKD reduces CIMT or the serum propensity for calcification or improves vitamin D metabolism. We hypothesized that lowering serum uric acid would increase 1,25(OH)_2_D, lower iPTH levels, and increase the expression of extra-renal 1α-hydroxylase. We found that, although allopurinol effectively lowered serum uric acid levels over a period of 12 weeks, it did not change CIMT or T_50_. Furthermore, we observed no significant change in vitamin D metabolites (including 1,25(OH)_2_D) or in iPTH. Although FGF-23 increased slightly in the allopurinol group, this was not statistically significant. Contrary to our hypothesis, the 12-week treatment with allopurinol did not lead to increased 1α-hydroxylase protein expression in the venous endothelial cells of a subset of participants. As such, our data indicate that uric acid-lowering does not significantly impact the systemic manifestations of CKD-MBD including vascular calcification or the expression of 1α- hydroxylase in extra-renal tissues.

Vascular calcification is a multi-faceted complication of CKD. Dysregulation of the mineral and bone axis plays a central role in the pathological shifts in normal mineralization between the bone and the vasculature in patients with CKD [35]. Restoration of active vitamin D and suppression of secondary hyperparathyroidism are hypothesized to restore normal mineralization at least partially [36]. As such, we hypothesized that improved vitamin D activation and reduced iPTH levels would lead to lower CIMT and higher T_50_. Uric acid-lowering had been shown to improve vitamin D metabolism in patients with CKD previously. In a prior study published by Vanholder *et al*. [37], serum 1,25(OH)_2_D increased from 30.8 ± 2.7 pg/mL to 38.2 ± 4.8 pg/mL (P < 0.01) after only 1 week treatment with 300 mg of allopurinol per day in patients with CKD. Our data contrast with these findings. One possible explanation for our findings is that our patients may have had inadequate 25(OH)D substrate. In both groups randomized to placebo and allopurinol, 25(OH)D levels were >25 ng/mL. While these values may be adequate in the absence of CKD [38], it is possible that higher levels of 25(OH)D (>30 ng/mL) are needed in CKD due to reduced nephron mass and impaired 1α-hydroxylase activity [39–41]. Consistent with this, in the study by Vanholder *et al*., baseline 25(OH)D levels were >50 ng/mL [37]. Thus, inadequate substrate may have influenced our findings.

Alternatively, it is likely that other factors (other than hyperuricemia) are predominantly responsible for reduced vitamin D activation in CKD, such as reduced nephron mass or increased FGF-23 [42]. If this is the case, lowering serum uric acid levels is likely to be of little or no benefit in patients with moderate CKD. The absence of significant improvement in the extra-renal expression of the 1α-hydroxylase enzyme in the treatment arm compared to placebo supports this conclusion. In the absence of increased enzyme expression, it is unlikely that enzyme activity would increase. Additionally, these data suggest that, rather than reduced nephron mass, circulating factors (such as FGF-23) in CKD represent the predominant regulator of this enzyme.

Previous data have suggested that higher FGF-23 levels associate with higher uric acid levels even in the absence of significant CKD [43, 44]. More recently, the potential association between uric acid and FGF-23 was evaluated in 537 patients with CKD in a cross-sectional analysis [45]. FGF-23 was independently associated with higher serum uric acid levels and with reduced urate clearance by the kidneys. While this data is limited by its cross-sectional nature, the authors suggested that FGF-23 may modulate urate metabolism in patients with CKD [45]. One proposed mechanism by which this may occur is secondary hyperparathyroidism. The rise in FGF-23 levels in CKD, suppresses vitamin D activation by the kidneys, leading to secondary hyperparathyroidism [46]. Hyperuricemia has long been noted in patients with primary hyperparathyroidism [47]. Although the exact mechanism by which this occurs remains unclear, earlier studies evaluating urate clearance in primary hyperparathyroidism suggest that tubular urate secretion is reduced via hypo-perfusion of the capillary network perhaps secondary to hypercalcemia [48]. Based on our study, we cannot determine whether secondary hyperparathyroidism contributes to hyperuricemia seen in CKD.

In addition to the CKD-MBD markers measured here, certain deficiencies have been shown to cause vascular calcification such as Klotho [49] and Fetuin-A deficiency [50]. Such deficiencies are not expectedly modified by uric acid and likely play an important role in vascular aging and calcification. As noted in the introduction, CKD-MBD is but one mechanism that contributes to vascular calcification in patients with CKD and certain processes such as local vascular inflammation, elastin degradation, and vascular smooth muscle osteogenic differentiation contribute to the disease [4–6]. Based on our previous analysis of the parent study, we found no evidence that lowering serum uric acid levels reduces systemic or vascular inflammation in CKD [23]. These data are consistent with our negative findings in the current analysis.

Our study has several limitations. First, this was a post-hoc analysis of the original study and the reported outcomes here were not pre-determined secondary end points. As such our findings should not be generalized. Second, regarding surrogate outcomes for vascular calcification, we were unable to measure all relevant markers (such Fetuin-A) and it is possible that longer study duration might have yielded different results. Third, as noted above, 25(OH)D levels were borderline adequate in our patients and we cannot exclude the possibility of inadequate substrate. Finally, while we were able to evaluate endothelial expression of 1α-hydroxylase protein, the sample size included was small. Notwithstanding these limitations, our study has several strengths including the ability to compare the treatment arm to placebo, the evaluation of CIMT and serum calcification propensity, the measurement of vitamin D metabolites by an experienced laboratory with standardized protocols, and the thorough evaluation of CKD-MBD parameters including the extra-renal expression of 1α-hydroxylase enzyme.

In conclusion, uric acid-lowering therapy does not improve CIMT, serum calcification propensity, nor vitamin D metabolism in patients with stage 3 CKD. Lowering serum uric acid levels did not improve the expression of 1α-hydroxylase in the endothelium of patients with CKD. Our data suggest that other factors play a more important role than uric acid in the vascular calcification of CKD-MBD.

## Abbreviations

CKD: Chronic kidney disease
MBD: Mineral and bone disorder
1,25(OH)_2_D: 1,25-dihydroxyvitamin D
iPTH: Intact parathyroid hormone
CIMT: Carotid intima-media thickness
CVD: Cardiovascular disease
FGF-23: Fibroblast growth factor-23
eGFR: Estimated glomerular filtration rate
MDRD: Modification of Diet in Renal Disease
CTRC: Clinical and Translational Research Center
CPPs: Calciprotein particles
HPLC: High-performance liquid chromatography
CKD-EPI: Chronic Kidney Disease Epidemiology Collaboration
DM: Diabetes Mellitus
